# Lung cancer immunotherapy in 2025: where we stand and what comes next?

**DOI:** 10.3389/fimmu.2025.1728163

**Published:** 2026-01-16

**Authors:** Jie Han, Zhibo Yang, Hai Zhao

**Affiliations:** 1 Department of Anesthesiology, Lanzhou University Second Hospital, Lanzhou, Gansu, China; 2 Department of Neurosurgery, 3201 Hospital of Xi’an Jiaotong University Health Science Center, Hanzhong, Shaanxi, China; 3 Department of Neurosurgery, The Affiliated Hospital of Qingdao University, Qingdao, Shandong, China

**Keywords:** biomarkers, CAR-T, combination therapy, immune checkpoint inhibitors, immunotherapy, lung cancer, novel modalities, resistance mechanisms

## Abstract

Lung cancer continues to be the leading cause of cancer-related mortality worldwide, accounting for more deaths than breast, colorectal, and prostate cancers combined. Over the past decade, the introduction of immunotherapy has reshaped treatment paradigms, offering hope for long-term survival in a disease historically associated with dismal outcomes. The incorporation of immune checkpoint inhibitors (ICIs) into the treatment of non-small cell lung cancer (NSCLC) and small cell lung cancer (SCLC) has improved outcomes across early-stage, locally advanced, and metastatic settings. However, only a fraction of patients derive durable benefit, and challenges remain in overcoming resistance, predicting response, managing toxicity, and ensuring equitable access. This review provides a comprehensive overview of current progress in lung cancer immunotherapy. It discusses the immunobiology of lung tumors, the role of checkpoint blockade across disease stages, mechanisms of resistance, biomarker development, and combination strategies. Emerging modalities, including bispecific antibodies, CAR- and TCR-based cellular therapies, natural killer (NK) cell platforms, cytokine agonists, oncolytic viruses, and vaccines, are explored in depth. We also evaluate the translational significance of preclinical models, toxicity management, and issues of equity and accessibility. Finally, we outline key future directions that may redefine lung cancer immunotherapy in the coming years. Collectively, these advances highlight a transition from broad applications of checkpoint inhibition toward stage-specific, biomarker-driven, and multimodal immunotherapy approaches designed to convert temporary responses into durable remissions and, ultimately, cures.

## Introduction

**Figure 1:**
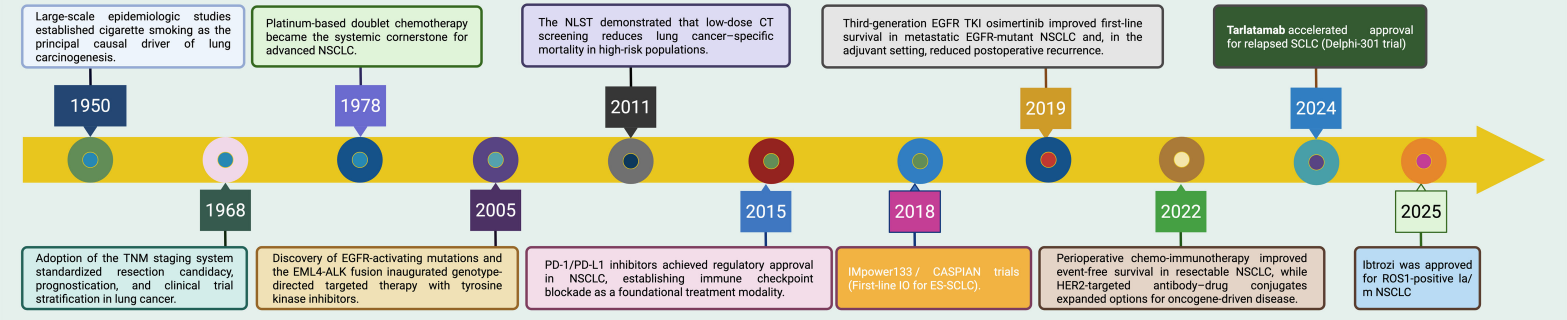
Evolution of lung cancer management and immunotherapy milestones (1950–2025). Landmark advances that have shaped lung cancer prevention, diagnosis, and systemic therapy from 1950 to 2025. Key inflection points include: (i) recognition of cigarette smoking as the principal causal driver of lung carcinogenesis (1950), catalyzing prevention policies; (ii) adoption of the TNM staging system to standardize resection candidacy, prognostication, and clinical-trial stratification (1968); (iii) establishment of platinum-based doublet chemotherapy as the systemic backbone for advanced NSCLC (1978); (iv) the NLST demonstration that low-dose CT screening reduces lung cancer–specific mortality in high-risk populations (2011); (v) discovery of EGFR-activating mutations and EML4-ALK fusion (2005), followed by third-generation EGFR TKI improving first-line survival and reducing postoperative recurrence (2019); (vi) regulatory approval of PD-1/PD-L1 inhibitors, establishing immune checkpoint blockade as a foundational treatment modality (2015); (vii) establishment of chemo-immunotherapy as the first-line standard for extensive-stage SCLC based on the IMpower133 and CASPIAN trials (2018); (viii) perioperative chemo-immunotherapy improving event-free survival in resectable NSCLC and expansion of HER2-directed antibody–drug conjugates (2022); and (ix) accelerated approval of tarlatamab for relapsed SCLC (2024), followed by the approval of Ibtrozi for ROS1-positive NSCLC (2025). NSCLC, non-small cell lung cancer; CT, computed tomography; NLST, National Lung Screening Trial; EGFR, epidermal growth factor receptor; ALK, anaplastic lymphoma kinase; TKI, tyrosine-kinase inhibitor; PD-1/PD-L1, programmed cell death-1/ligand-1; ICI, immune checkpoint inhibitor; HER2, human epidermal growth factor receptor 2; ADC, antibody–drug conjugate; EFS, event-free survival.

Lung cancer represents a global health crisis, with approximately 2.2 million new cases and 1.8 million deaths annually (1–3). Despite significant improvements in early detection and surgical techniques, the majority of patients are diagnosed at advanced stages, where prognosis remains poor. For decades, chemotherapy was the mainstay of treatment, and although targeted therapies brought improvements for genetically defined subgroups such as EGFR- or ALK-positive NSCLC, the survival benefit was often limited by the inevitable development of resistance (4–7). The introduction of immunotherapy, particularly ICIs targeting the PD-1/PD-L1 axis, marked a paradigm shift (8, 9). These therapies harness the immune system’s ability to recognize and attack tumor cells, leading to long-term remissions in subsets of patients who would previously have faced inevitable progression (**Figure 1**).

However, enthusiasm must be tempered by realism. The majority of lung cancer patients either do not respond to immunotherapy initially or develop resistance after an initial response (10). Additionally, certain subtypes such as EGFR- or ALK-driven tumors, as well as the majority of SCLC, remain largely refractory to current ICIs (11, 12). Furthermore, disparities in access to immunotherapy across countries and within populations underscore the urgent need for strategies that are not only biologically innovative but also widely accessible. Against this backdrop, lung cancer serves as a model for understanding how to best apply immunotherapy in solid tumors: it highlights both the remarkable potential and the persistent barriers of immune-based treatment.

This review aims to provide a comprehensive synthesis of the state of lung cancer immunotherapy until 2025. Unlike previous reviews that largely catalog the history of checkpoint inhibition, this article uniquely addresses the critical inflection point of 2025: the transition from broad, ‘one-size-fits-all’ immunotherapies toward stage-specific, biomarker-driven, and multimodal strategies. We specifically highlight the integration of emerging modalities (such as DLL3-targeting agents and TILs), the modulation of the microbiome, and the urgent need to address global equity in the era of precision immuno-oncology. It begins by discussing the immunobiology of lung tumors and how these features shape responsiveness to therapy. It then reviews clinical advances with checkpoint blockade across metastatic, locally advanced, and early-stage disease, followed by a discussion of resistance mechanisms and biomarkers that can refine patient selection. Subsequent sections focus on combination strategies, emerging immunotherapeutic modalities beyond ICIs, preclinical and translational models, toxicity management, and considerations of equity and regulation. The final sections highlight future directions, emphasizing the need for biomarker-driven, multimodal, and globally accessible strategies.

## Immunobiology of lung tumors

The immune landscape of lung cancer is defined by both its origins and its microenvironment. Unlike many other solid tumors, lung cancers frequently arise in tissues chronically exposed to carcinogens such as tobacco smoke and environmental pollutants. This exposure results in an exceptionally high tumor mutational burden (TMB), which theoretically generates abundant neoantigens that could make lung cancer highly immunogenic (13). Yet paradoxically, lung tumors often evolve mechanisms that allow them to escape immune detection and destruction.

A central feature of lung cancer immunobiology is defective antigen presentation (14). Many NSCLCs demonstrate downregulation of HLA class I molecules, mutations in beta-2 microglobulin, or loss of heterozygosity in HLA loci, all of which impair T-cell recognition (15–17). Moreover, certain oncogenic drivers directly influence immune evasion. For example, STK11 and KEAP1 co-mutations create a non-inflamed, metabolically hostile tumor microenvironment (TME), while MYC amplifications may upregulate PD-L1 and drive T-cell exhaustion (18, 19).

**Figure 2:**
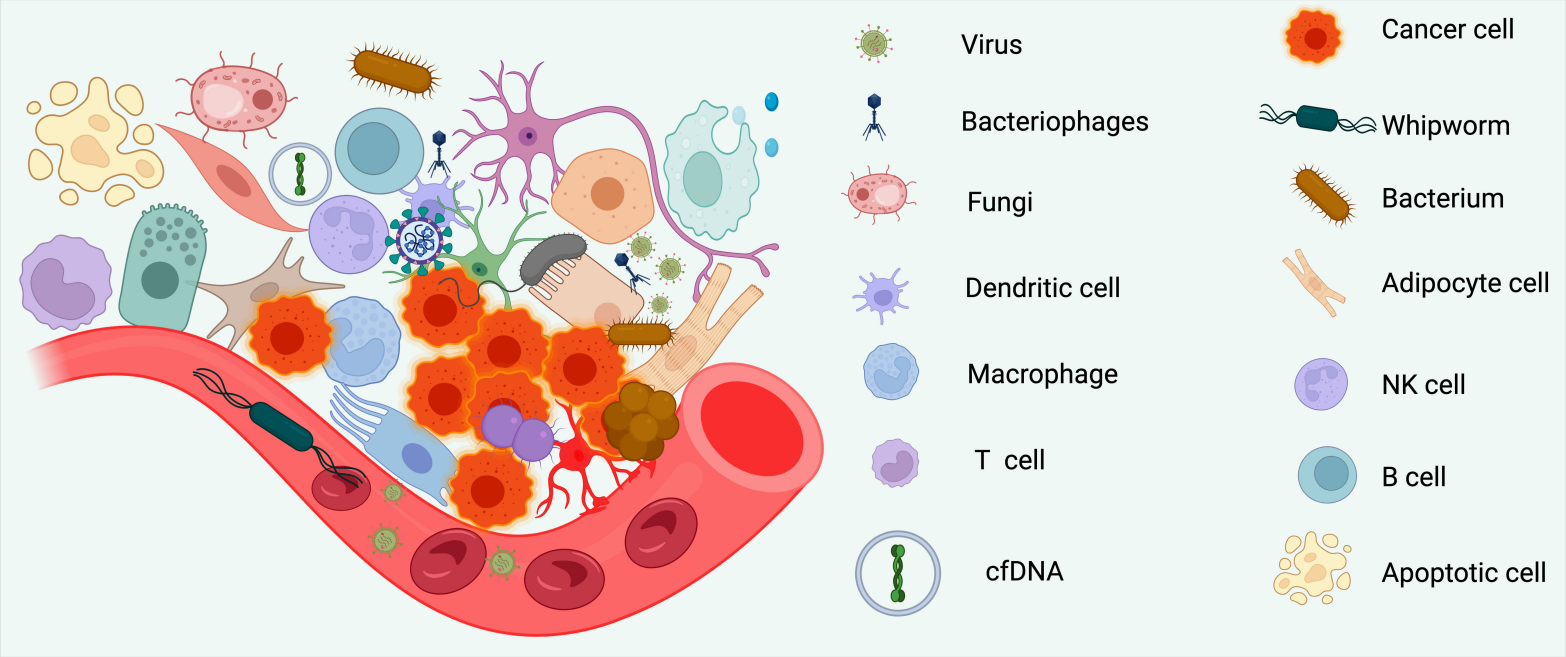
Circulating and tumor-associated components shaping the lung cancer immune microenvironment. Schematic overview of cellular and acellular elements that influence antitumor immunity at the tumor–vasculature interface. The left panel depicts a tumor focus adjacent to a blood vessel with infiltrating and resident immune populations, stromal elements, and microbial signals. Innate and adaptive leukocytes include dendritic cells, macrophages, T cells, B cells, and natural killer (NK) cells. Non-immune cellular constituents—adipocytes, apoptotic bodies, and neural/glial elements—contribute metabolic and paracrine cues that modulate antigen presentation and cytotoxic function. Extrinsic biological inputs comprise viruses, bacteriophages, fungi, helminth components (whipworm), and bacteria, which collectively represent the tumor–tissue–microbiome axis and may alter local pattern-recognition signaling and cytokine tone. Cell-free DNA (cfDNA) in the circulation provides a measurable surrogate of tumor burden and clonal dynamics. The right panel lists visual icons corresponding to the elements represented within the tumor bed and bloodstream. The illustration emphasizes the convergence of microbial, stromal, immune, and tumor-intrinsic factors that jointly determine response or resistance to immunotherapy. NK, natural killer; cfDNA, cell-free DNA.

The TME in lung cancer is often characterized by immunosuppressive cell populations, including tumor-associated macrophages (TAMs), myeloid-derived suppressor cells (MDSCs), and regulatory T cells (Tregs) (20–24). These cells release cytokines such as IL-10 and TGF-β, which inhibit effector T-cell function. Abnormal tumor vasculature and dense stroma restrict immune infiltration, while metabolic constraints such as hypoxia and lactate accumulation further impair cytotoxic T-cell activity (25–28) (**Figure 2**).

Comparisons between NSCLC and SCLC underscore the diversity of immune contexts (29). While NSCLC displays heterogeneous PD-L1 expression and varying immune infiltration, SCLC is generally immunologically “cold,” characterized by rapid proliferation, genomic instability, and low antigen presentation (30, 31). This difference helps explain why SCLC has derived less durable benefit from ICIs, despite its high mutational load. Understanding these immunobiological features provides the foundation for rational development of therapies that seek not only to release immune inhibition but also to actively remodel the TME.

## Oncogenic drivers and immune phenotypes in NSCLC

A more granular understanding of the genomic landscape of NSCLC has revealed that recurrent oncogenic drivers not only define therapeutic opportunities for targeted agents but also profoundly shape the immune phenotype and response to checkpoint blockade (32–34). Canonical actionable alterations in NSCLC include mutations in EGFR and BRAF (most commonly V600E), rearrangements involving ALK, ROS1, RET, and NTRK, MET exon 14 skipping, and ERBB2/HER2 insertions (35–40). These lesions tend to arise in never- or light-smokers, are frequently associated with a relatively lower tumor mutational burden, and often coexist with an immune-excluded or non-inflamed tumor microenvironment. Clinically, EGFR- or ALK-driven tumors derive limited and inconsistent benefit from current PD-1/PD-L1 inhibitors, and immune-related toxicities can be accentuated when ICIs are given before or concurrently with tyrosine kinase inhibitors. By contrast, KRAS-mutant NSCLC—especially in the setting of a smoking-related mutational signature—typically harbors higher TMB and can display more inflamed immune infiltration, although the co-mutation pattern is critical for determining outcome (41).

Among KRAS-mutant tumors, co-alterations in STK11 (LKB1) and KEAP1 generally define an immunologically ‘cold’ phenotype. Mechanistically, these mutations create a metabolically hostile TME characterized by blunted STING-mediated interferon signaling and the accumulation of immunosuppressive metabolites such as adenosine and lactate. This results in defective antigen presentation and the exclusion of cytotoxic T cells, rendering these tumors largely refractory to PD-1 blockade. Similarly, genetic disruptions in antigen presentation machinery, such as B2M loss of heterozygosity, directly compromise MHC class I recognition, serving as a foundational barrier to immune surveillance (42, 43).

Other genomic features intersect with immune control in more nuanced ways. Loss of heterozygosity at HLA loci or truncating mutations in B2M directly compromise MHC class I antigen presentation, predisposing to primary or acquired resistance even in tumors with nominally high TMB. Amplification or overexpression of MYC can upregulate PD-L1 and drive T-cell exhaustion programs, while alterations in STK11/KEAP1 and other metabolic regulators remodel nutrient availability and redox balance within the tumor bed (44). Collectively, these data highlight that NSCLC genomics and immunobiology are tightly coupled: actionable drivers define not only eligibility for targeted therapy but also baseline immunogenicity, the architecture of the tumor microenvironment, and the likelihood of durable benefit from immune checkpoint blockade. Integrating driver status and co-mutation patterns into immunotherapy decision-making—rather than treating them as separate domains—will be essential for truly precision lung cancer care.

## Immune checkpoint blockade across the disease continuum

Immune checkpoint blockade (ICB) now spans the lung cancer continuum, from first-line therapy in metastatic NSCLC to consolidation after chemoradiation in unresectable stage III disease and perioperative regimens that improve event-free survival in resectable tumors; in extensive-stage SCLC, ICB combined with chemotherapy has become a foundational backbone. Across settings, rational combinations—anti-VEGF priming, chemoradiation sequencing, and emerging dual-checkpoint strategies (e.g., PD-1/LAG-3, PD-1/TIGIT)—seek to convert immune-excluded phenotypes and deepen durability (45). Patient selection is increasingly guided by integrated biomarkers that move beyond PD-L1 alone, incorporating ctDNA/MRD trajectories, spatial immune architecture (e.g., TLS), antigen-presentation integrity, and radiomic correlates. In terms of research progress: metastatic NSCLC has matured from PD-(L)1 monotherapy to chemo-IO backbones and selected dual-IO strategies; locally advanced NSCLC has established durable overall-survival gains with post-CRT consolidation ICB; resectable NSCLC now benefits from neoadjuvant chemo-IO and perioperative approaches with reproducible improvements in pCR/MPR and EFS (**Table 1**); and in SCLC, chemo-ICB has produced modest but significant survival gains with ongoing trials exploring maintenance, biomarker-enriched cohorts, and next-generation checkpoints.

**Table 1:** Clinical trials of ICIs in combination with immunotherapy in NSCLC.

Clinical trial	Target or combination regimen	Setting	Intervention	Outcome (months)
CheckMate 227	PD-1, CTLA-4	Advanced NSCLC	Arm A:nivolumab+ ipilimumab Arm B:nivolumab	mOS:17.1 vs.15.7; mPFS:5.1 vs.4.2
NCT03563716	PD-L1, TIGIT	Advanced or metastatic NSCLC	Arm A:Tiragolumab + atezolizumab; Arm B:Placebo + atezolizumab	mPFS:5.4 vs.3.6
NCT02352948	PD-1	Advanced or metastatic NSCLC (Stage IIIB-IV)	Arm A:Durvalumab; Arm B:Durvalumab+ tremelimumab	mOS:11.7 vs.11.5; mPFS:3.8 vs.3.5
NCT03302234	PD-1, CTLA-4	Untreated metastatic NSCLC	Arm A:Pembrolizumab+ ipilimumab; Arm B: Pembrolizumab+placebo	mOS:21.4 vs.21.9; mPFS:8.2 vs.8.4
NCT02453282	PD-L1, CTLA-4	NSCLC	Arm A:Durvalumab+tremelimumab; Arm B: Durvalumab	mOS:11.9 vs.16.3;

NCT, National Clinical Trial; mOS, median overall survival; mPFS,median progression-free survival; NSCLC, non-small cell lung cancer; PD-1, prog rammed cell death-1; PD-L1, programmed cell death ligand 1; CTLA-4, cytotoxic T lymphocytes cell antigen-4; TIGIT, T cell immunoglobulin and ITIM domain.

### Metastatic NSCLC

Checkpoint inhibitors revolutionized the treatment of metastatic NSCLC. In patients with PD-L1 expression ≥50%, single-agent PD-1 inhibitors achieve durable responses, with a subset experiencing long-term survival beyond five years (46–48). For patients with lower PD-L1 expression or without a defined biomarker, combination strategies with chemotherapy remain the backbone of therapy (49). Chemo-immunotherapy enhances tumor antigen release and promotes dendritic cell activation, thereby synergizing with ICIs (50). For instance, in the pivotal KEYNOTE-189 trial (non-squamous NSCLC), the addition of pembrolizumab to chemotherapy reduced the risk of death by approximately 50% (HR = 0.49) compared to chemotherapy alone (51). Similarly, in squamous NSCLC (KEYNOTE-407), the combination regimen demonstrated a significant survival benefit (median OS: 17.1 vs. 11.6 months) (52).

Dual checkpoint blockade combining PD-1 and CTLA-4 inhibitors has emerged as another viable option, offering chemotherapy-free regimens for selected patients (8). While toxicity is greater, some patients achieve long-lasting responses. Importantly, these strategies highlight that ICIs are not simply adjuncts but central components of metastatic NSCLC therapy. Nevertheless, challenges remain, including the management of immune-related adverse events (irAEs), identification of predictive biomarkers, and understanding mechanisms of resistance (53).

### Locally advanced NSCLC

In unresectable stage III NSCLC, the PACIFIC trial was practice-changing. Consolidation durvalumab after chemoradiotherapy significantly prolonged survival, with sustained benefit observed at five years, demonstrating a robust 5-year overall survival rate of 42.9% in the durvalumab arm versus 33.4% in the placebo arm (54–56). This strategy demonstrated that ICIs could improve outcomes in patients treated with curative intent, not only in the metastatic setting. Ongoing studies are investigating whether concurrent administration of ICIs with chemoradiotherapy can further enhance efficacy, although safety remains a key concern. Novel regimens exploring intensified consolidation or the addition of other immunomodulatory agents are also underway (57).

The success of PACIFIC underscores a broader principle: earlier integration of immunotherapy may yield greater benefit. By engaging the immune system when tumor burden is lower and immune competence is relatively preserved, the likelihood of achieving durable remission increases (**Table 2**).

**Table 2:** Summary of ICI-based combination strategies for advanced non–small cell lung cancer.

Study	Phase	Sample size	Histology	Median PFS	Median OS	Treatment arms	PD-L1
NCT02039674	II	123	non- squamous	20.0 vs.9.3	NR vs.21.1	Pembrolizumab/PC vs.carboplatin/pemetrexed	Nope
NCT02578680	III	616	non- squamous	9.0 vs.4.9	22.0 vs.10.7	Pembrolizumab/PC vs. carboplatin/pemetrexed	Nope
NCT02775435	III	559	squamous	8.0 vs. 5.1	17.1 vs.11.6	Pembrolizumab/ chemotherapy vs. carboplatin/(nab-)paclitaxel	Nope
NCT02367781	III	723	non- squamous	7.0 vs.5.5	18.6 vs.13.9	Atezolizumab/CnP vs. carboplatin/nab-paclitaxel	Nope
NCTO236779	III	683	squamous	6.5 vs. 5.6	14.6 vs.14.3	Atezolizumab/CnP vs. carboplatin/nab-paclitaxel	Nope
NCT02657434	III	578	non- squamous	7.6vs.5.2	18.1 vs.13.6	Atezolizumab/PC vs. pemetrexed-carboplatin/cisplatin	Nope
NCT02366143	III	1202	non- squamous	8.3 vs.6.8	19.2 vs.14.7	Atezolizumab/BCP vs. bevacizumab/carboplatin/paclitaxel	Nope
NCT02477826	III	1739	squamous and non- squamous	7.2 vs.5.5	17.2 vs.12.2	Nivolumab/pilimumab vs.chemotherapy	Nope
NCT02453282	III	1118	squamous and non- squamous	3.9 vs.5.4	11.9 vs.12.9	Durvalumab/Tremelimumab vs.chemotherapy	PD-L1≥25%

BCP, bevacizumab + carboplatin + paclitaxel; CnP, carboplatin + nab-paclitaxel;ICI, immune checkpoint inhibitor; NSCLC, non–small cell lung cancer (from table title);OS, overall survival (months);PC, platinum–pemetrexed chemotherapy (carboplatin/cisplatin + pemetrexed);PD-L1, programmed death-ligand 1 (tumor expression);PFS, progression-free survival (months);vs., versus (used for arm-to-arm comparisons).

### Resectable NSCLC

The extension of ICIs into resectable NSCLC is one of the most exciting developments in thoracic oncology (58, 59). Neoadjuvant immunotherapy, particularly when combined with chemotherapy, significantly increases major pathologic response and complete response rates compared with chemotherapy alone (60, 61). Trials have also demonstrated improvements in event-free survival without compromising the feasibility or safety of surgery. Perioperative strategies, in which patients receive both neoadjuvant and adjuvant immunotherapy, appear especially promising.

Adjuvant immunotherapy after complete resection is another strategy supported by randomized trials (62, 63). In this setting, ICIs improve disease-free survival, particularly in patients with higher PD-L1 expression. Together, these approaches signal a future in which immunotherapy is a standard component of multimodality treatment for early-stage lung cancer, raising the possibility of significantly increasing cure rates.

Perioperative immunotherapy in resectable NSCLC is typically delivered in fixed durations irrespective of residual risk. A ctDNA-minimal residual disease (MRD)–guided algorithm can transform this paradigm into a dynamic, data-driven program (64–66). Pre-treatment stratification combines ctDNA positivity, PD-L1 status, and T-cell clonotypic diversity to determine whether to augment neoadjuvant therapy with micro-dose radiation or intratumoral priming (e.g., oncolytic vectors) to ignite local antigen presentation. Post-resection, MRD conversion to negativity across consecutive time points supports de-escalation of adjuvant immunotherapy, whereas MRD persistence or re-emergence triggers short-course intensification with mechanism-complementary agents (e.g., adenosine pathway antagonists or anti-VEGF add-on) selected to minimize overlapping pulmonary toxicity (67, 68). For patients with low-level, fluctuating MRD without radiographic relapse, low-toxicity immunometabolic modulation can serve as a holding strategy to prevent overtreatment while preserving immune fitness. Embedded correlative studies should tie MRD kinetics to spatial immune remodeling, establishing trajectory-based stopping rules that move beyond categorical PD-L1 thresholds (65, 69). This adaptive construct reframes perioperative immunotherapy as precision maintenance of immunologic control, aligning treatment intensity with evolving relapse biology.

### SCLC

Small cell lung cancer, although less common, remains one of the most lethal malignancies. The addition of ICIs to first-line chemotherapy has modestly improved survival in extensive-stage SCLC, establishing new standards of care (70, 71). The addition of ICIs to first-line chemotherapy has modestly improved survival in extensive-stage SCLC, establishing new standards of care. The IMpower133 trial reported a median OS of 12.3 months with atezolizumab plus chemotherapy versus 10.3 months with chemotherapy alone, while the CASPIAN trial showed similar benefits with durvalumab (median OS: 13.0 vs. 10.3 months). However, durable responses remain rare, and resistance is nearly universal (72–74). However, durable responses remain rare, and resistance is nearly universal. Unlike NSCLC, PD-L1 expression is generally low in SCLC, and the biology of the disease is dominated by rapid progression and profound immune evasion (75, 76).

Recent advances have introduced new targets for SCLC, such as DLL3, which is highly expressed in neuroendocrine tumors (77). The development of bispecific T-cell engagers and CAR-T cells targeting DLL3 provides new avenues of exploration (78–80). While early clinical data are promising, more research is needed to translate these therapies into durable benefits. The SCLC landscape illustrates the urgency of innovation beyond PD-1/PD-L1 blockade.

**Figure 3:**
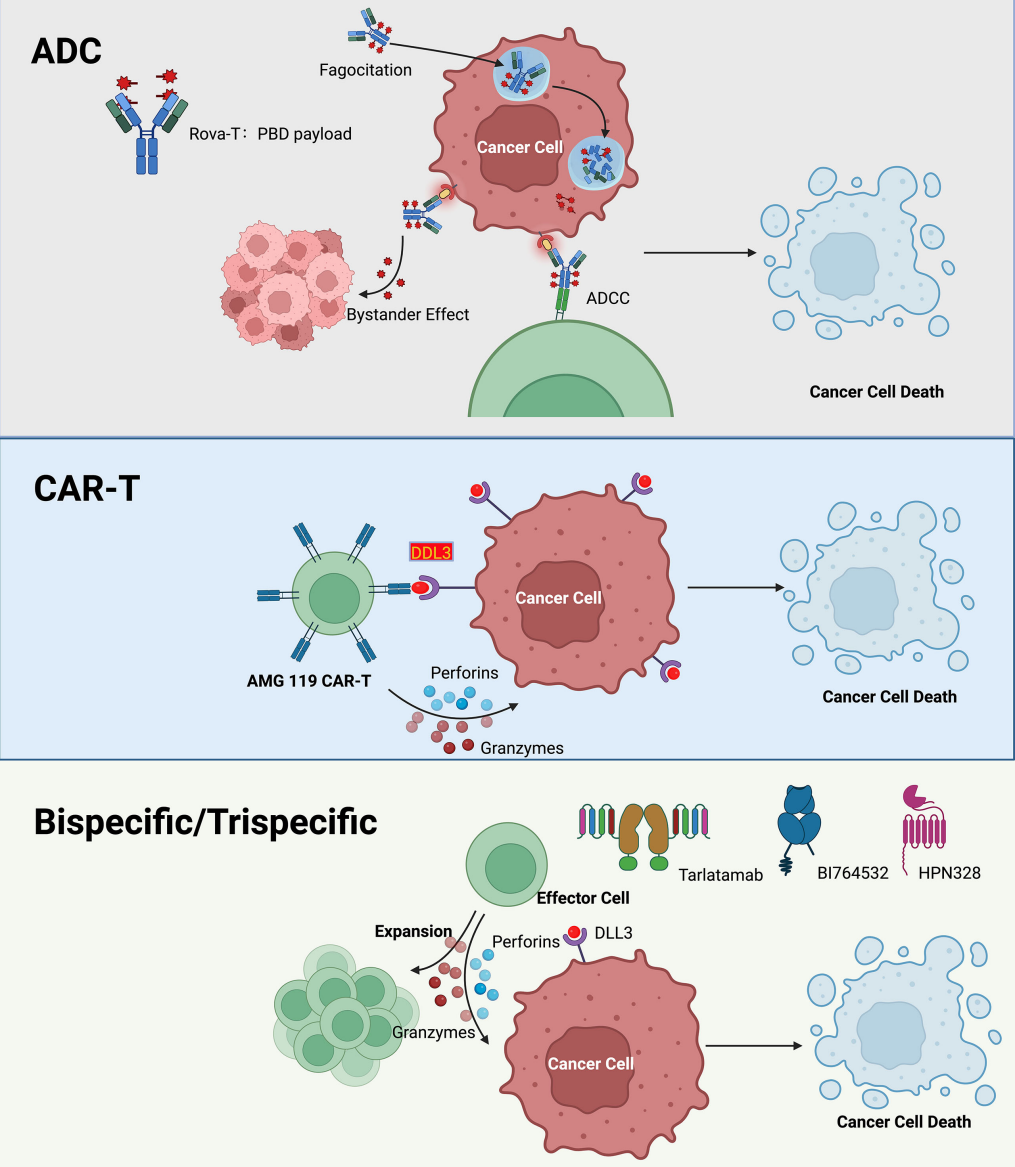
Mechanisms of action of the various novel therapies targeting DLL3. Schematic comparison of three targeted immunotherapeutic modalities directed at DLL3-positive tumor cells. Top (ADC): An antibody–drug conjugate (example: rovalpituzumab tesirine, Rova-T) binds cell-surface DLL3, undergoes internalization, and releases a cytotoxic pyrrolobenzodiazepine (PBD) payload that induces DNA damage and apoptosis; Fc-mediated effector functions enable antibody-dependent cellular cytotoxicity (ADCC) and a bystander effect on adjacent DLL3-low cells. Middle (CAR-T): Autologous T cells engineered with a DLL3-specific chimeric antigen receptor (e.g., AMG 119) recognize tumor targets and mediate perforin/granzyme-dependent lysis, leading to cancer cell death. Bottom (Bispecific/Trispecific): T-cell–redirecting antibodies (e.g., tarlatamab, BI 764532, HPN328) simultaneously engage DLL3 on tumor cells and CD3 (± additional co-receptors) on effector lymphocytes, promoting synapse formation, T-cell expansion, and cytotoxicity via perforins and granzymes. Together, these platforms illustrate complementary mechanisms to exploit DLL3 for therapeutic targeting in small-cell lung cancer and other DLL3-expressing thoracic malignancies. ADC, antibody–drug conjugate; ADCC, antibody-dependent cellular cytotoxicity; CAR-T, chimeric antigen receptor T cell; DLL3, delta-like ligand 3; PBD, pyrrolobenzodiazepine.

DLL3-directed T-cell engagers have created a new therapeutic avenue in relapsed small-cell lung cancer; however, antigen down-modulation and lineage plasticity threaten the durability of benefit (81). We propose a programmable antigen-switching framework in which surveillance assays and pre-specified treatment pivots are defined at therapy initiation. Longitudinal profiling of circulating tumor DNA and circulating tumor cells is used to monitor DLL3 expression and neuroendocrine state. Prespecified alerts—such as declining DLL3 signal, emergence of alternative targetable antigens, or transition to non-neuroendocrine phenotypes—trigger a planned secondary redirection to a validated alternative target, for example CEACAM5 or other SCLC-associated antigens, before full immune escape occurs (82, 83). This approach is supported by time-limited immunometabolic maintenance to preserve T-cell fitness between redirections, such as low-toxicity antagonism of the adenosine pathway, and by bone-marrow–sparing scheduling to minimize cumulative cytopenias. In contrast to the traditional sequence of progression followed by salvage therapy, antigen switching conceptualizes SCLC as a moving immune target and maintains T-cell pressure through serial yet coordinated retargeting. Programs based on this strategy should prospectively define quantitative switching thresholds, assay turnaround expectations, and safety stopping rules to enable reproducible implementation across multicenter settings (**Figure 3**).

## Resistance mechanisms

Despite the success of ICIs, most lung cancer patients do not achieve durable benefit, making resistance a central challenge. Resistance can be categorized as primary, where tumors never respond, or acquired, where tumors progress after initial benefit (84–86).

Primary resistance often stems from the absence of pre-existing T-cell infiltration, sometimes described as an “immune desert” (87). Primary resistance often stems from an ‘immune desert’ phenotype. As detailed in the Immunobiology section, specific genomic drivers like STK11/KEAP1 co-mutations orchestrate this non-inflamed state, preventing initial T-cell infiltration (88, 89). Defective antigen presentation due to HLA class I downregulation or beta-2 microglobulin mutations also contributes (90).

Acquired resistance involves adaptive changes within the tumor and TME (91). Tumors may develop mutations in interferon signaling pathways (e.g., JAK1/2), lose target antigens through clonal evolution, or upregulate alternative checkpoints such as TIM-3, LAG-3, and TIGIT. The TME may also shift toward greater immunosuppression, with expansion of Tregs and myeloid cells (92–95) (**Table 3**).

**Table 3:** Summary of emerging immunotherapeutic modalities and representative clinical trials in lung cancer.

Modality	Agent	Target	Mechanism of action	Key clinical trials / status
BiTEs	Tarlatamab (AMG 757)	DLL3 × CD3	Redirects cytotoxic T cells to lyse DLL3-expressing SCLC cells independent of MHC	Approved; DeLLphi-301 (NCT05060016)
	BI 764532	DLL3 × CD3	Induces T-cell-mediated killing of DLL3+ tumor cells	Phase I/II (NCT04429087)
	HPN328	DLL3 × CD3 × Albumin	Trispecific T-cell engager with extended half-life	Phase I/II (NCT04471727)
	Amivantamab	EGFR × MET	Targets EGFR/MET mutations and induces ICDA (Trogocytosis)	Approved; PAPILLON (NCT04538664)
CAR-T Cell	AMG 119	DLL3	Autologous T cells engineered with DLL3-specific chimeric antigen receptor.	Phase I
	TnMUC1 CAR-T	TnMUC1	Targets tumor-associated glycosylated MUC1 antigen	Phase I
	Anti-Mesothelin CAR-T	Mesothelin	Targets mesothelin-expressing solid tumors	Phase I/II
ADCs	HER2-directed ADCs	HER2	Delivers cytotoxic payload (topoisomerase I inhibitor) to HER2+ cells	Approved (HER2-mutant NSCLC); DESTINY-Lung01/02
	Rovalpituzumab tesirine	DLL3	Delivers PBD dimer toxin to DLL3+ cells	Discontinued (TAHOE/MERU trials)
Checkpoints	Tiragolumab	TIGIT	Blocks TIGIT-PVR interaction to restore CD226-mediated costimulation.	Phase III (SKYSCRAPER-01)
	Relatlimab / Favezelimab	LAG-3	Blocks LAG-3/MHC-II interaction to reinvigorate exhausted T cells.	Phase I/II
Cancer Vaccines	mRNA Vaccines	Neoantigens	mRNA encoding tumor-specific antigens to prime de novo T-cell responses^14^.	Phase I

BiTE, Bispecific T-cell Engager;ADC, Antibody-Drug Conjugate;CAR-T, Chimeric Antigen Receptor T-cell; DLL3, Delta-like Ligand 3; EGFR, Epidermal Growth Factor Receptor; HER2, Human Epidermal Growth Factor Receptor 2; LAG-3, Lymphocyte-Activation Gene 3; MET, Mesenchymal-Epithelial Transition Factor; MHC, Major Histocompatibility Complex; NSCLC, Non-Small Cell Lung Cancer; ICDA, immune cell-directed activity PBD, Pyrrolobenzodiazepine; SCLC, Small Cell Lung Cancer; TIGIT, T-cell Immunoreceptor with Ig and ITIM domains.

Metabolic resistance mechanisms are increasingly recognized. Tumors create nutrient-depleted environments, accumulate immunosuppressive metabolites such as adenosine and lactate, and exploit hypoxia-induced pathways to impair T-cell function (96, 97). These insights suggest that overcoming resistance requires not only more potent checkpoint blockade but also interventions that remodel the TME, restore antigen presentation, and alleviate metabolic constraints.

**Figure 4:**
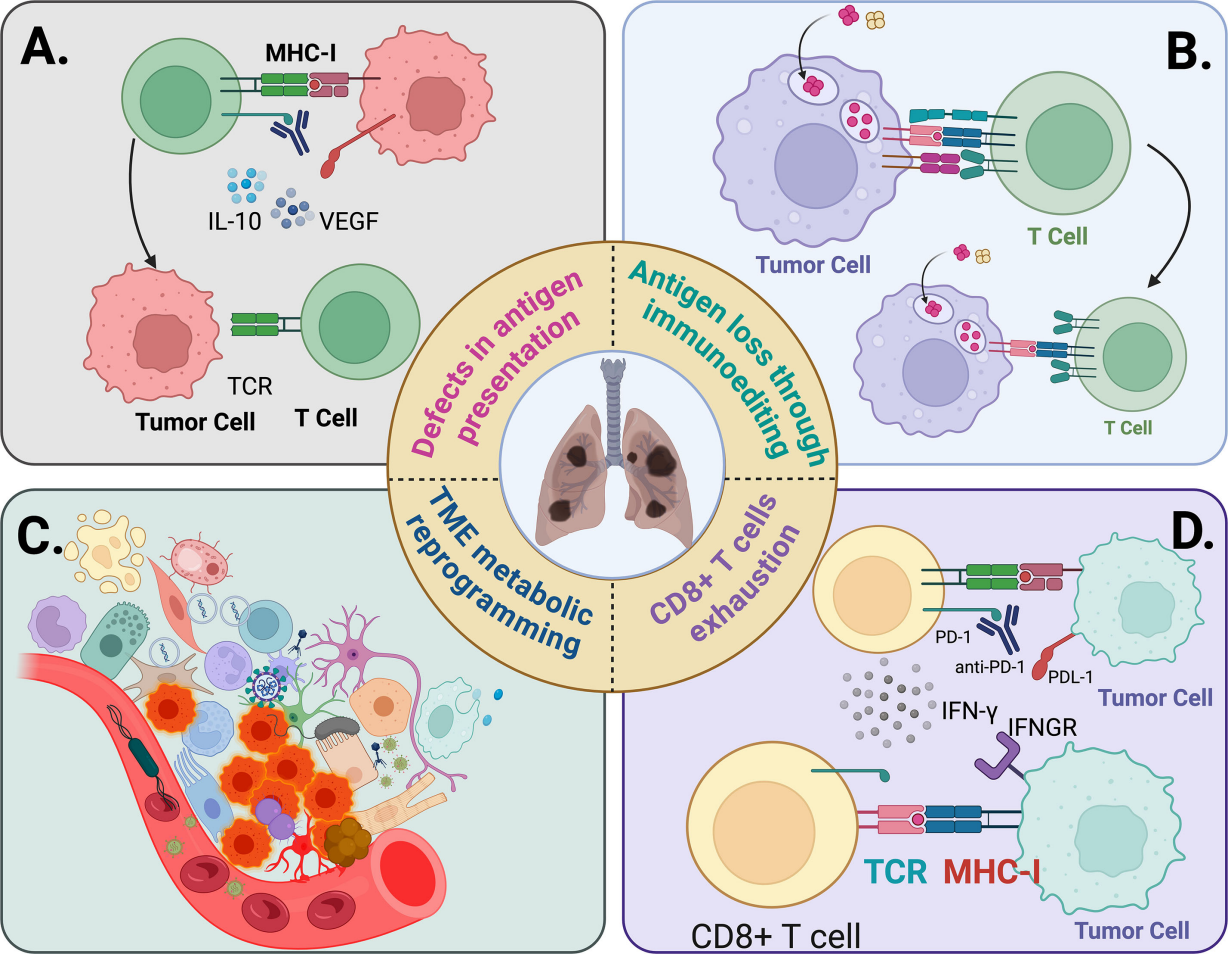
Schematic overview of mechanistic circuits that confer resistance to cancer immunotherapy. **(A)** Antigen processing and presentation failure. Disruptions across the MHC class I pathway—loss of heterozygosity or downregulation of HLA-A/B/C, beta-2 microglobulin truncations, TAP/TAPBP defects, and impaired proteasomal processing—impede generation and display of peptide–MHC complexes. As a result, cognate T-cell receptor engagement is curtailed despite checkpoint inhibition, yielding ineffective effector recruitment and immune escape under ICI therapy. **(B)** Inadequate tumor antigenicity. Low neoantigen burden, clonal dilution, or immunoediting-driven antigen loss diminishes the visibility of malignant cells to the adaptive immune system. Epigenetic silencing of cancer–testis antigens and defective IFN-γ signaling further reduce inducible antigen expression, lowering the probability of productive T-cell priming and favoring primary resistance. **(C)** Tumor-microenvironmental immunosuppression. Myeloid-dominant ecosystems (M2-skewed macrophages, MDSCs), regulatory T cells, and cancer-associated fibroblasts establish metabolic and physical barriers to infiltration and killing. Hypoxia, aberrant vasculature, and accumulation of adenosine and lactate suppress T-cell fitness; IDO1-mediated tryptophan catabolism and TGF-β/VEGF signaling reinforce exclusionary stroma and anti-inflammatory tone. These convergent stromal, metabolic, and cytokine cues blunt effector trafficking and function, driving both primary and adaptive resistance to immune checkpoint blockade. **(D)** CD8^+^ T-cell exhaustion and signaling refractoriness. Chronic antigen exposure and inflammatory stress establish an epigenetically fixed exhausted state characterized by elevated inhibitory receptors (PD-1, TIM-3, LAG-3, TIGIT), TOX/NR4A-driven transcriptional programs, and curtailed cytotoxic function with attenuated cytokine output (e.g., IFN-γ). Concurrent lesions in the IFN-γ–JAK/STAT axis or downstream antigen-presentation machinery can render tumor cells nonresponsive to reinvigorated T cells, limiting ICI benefit even when PD-1/PD-L1 is blocked.

Targeting these barriers requires a mechanism-anchored approach. For the STK11/KEAP1-driven ‘cold’ phenotype previously described, a synthetic-rescue strategy is proposed to treat this genotype not merely as a resistance marker, but as a distinct immunometabolic disorder requiring modular re-wiring (42). Rather than incremental add-ons to PD-(L)1 monotherapy, a synthetic-rescue strategy treats this genotype as an immunometabolic disorder requiring modular re-wiring. A stepwise approach can be prospectively evaluated: Step 1—Perfusion repair through anti-VEGF–mediated vascular normalization to reduce hypoxia and allow lymphocyte entry (98); Step 2—Myeloid reprogramming using CSF1R or PI3K-γ axis inhibitors to shift TAM polarization and restore dendritic-cell licensing (99); Step 3—Metabolic disinhibition with A2A/A2B antagonists to relieve adenosine-mediated T-cell suppression (100); Step 4—Selective oncogene targeting (e.g., KRAS G12C inhibition where applicable) in offset sequencing to avoid hepatotoxic synergy and to maintain neoantigen exposure during immune reinvigoration (101). Early success metrics should include spatial relief of exclusion (multiplex imaging), restoration of antigen-presentation signatures, and ctDNA reduction despite stable radiology, acknowledging that biologic wins may precede anatomic shrinkage. This framework upgrades “difficult-to-treat genomics” from a negative predictor into a mechanism-anchored treatment blueprint (**Figure 4**).

## Biomarkers of immunotherapy response

### PD-L1 expression

PD-L1 expression by immunohistochemistry remains the most widely used biomarker in NSCLC (102). High PD-L1 expression correlates with improved response rates and survival with single-agent ICIs, guiding clinical decisions in the metastatic setting (49). However, limitations abound. PD-L1 expression is highly heterogeneous within and between tumors, and temporal changes occur over the disease course (103). Furthermore, differences among assay platforms and antibody clones complicate interpretation. While PD-L1 testing provides useful guidance, it is insufficient as a stand-alone biomarker, particularly in predicting long-term benefit.

### Tumor mutational burden

TMB reflects the number of somatic mutations per megabase of DNA. Higher TMB is associated with increased neoantigen generation, theoretically enhancing immunogenicity (13, 104). Studies have shown correlations between high TMB and improved response to ICIs in some contexts, leading to regulatory approval of TMB as a tumor-agnostic biomarker in certain settings (105). Yet, in lung cancer, its predictive value remains inconsistent. Variability in sequencing platforms, cutoff definitions, and the influence of co-mutations limit its reliability. TMB is best considered as one component of a multifaceted biomarker strategy rather than a solitary predictor.

### Gene signatures and spatial biomarkers

Gene expression signatures reflecting interferon-γ signaling, cytotoxic T-cell infiltration, and immune-inflamed phenotypes have been associated with improved outcomes (87, 106). Spatial biomarkers, including the presence of tertiary lymphoid structures, provide further insight into the architecture of immune infiltration (107). TLSs serve as local sites of antigen presentation and T-cell priming, and their presence correlates with favorable ICI responses independent of PD-L1 expression. Spatial profiling technologies, including multiplex immunohistochemistry and spatial transcriptomics, are advancing the ability to identify predictive immune niches (108–110).

### Circulating biomarkers

**Figure 5:**
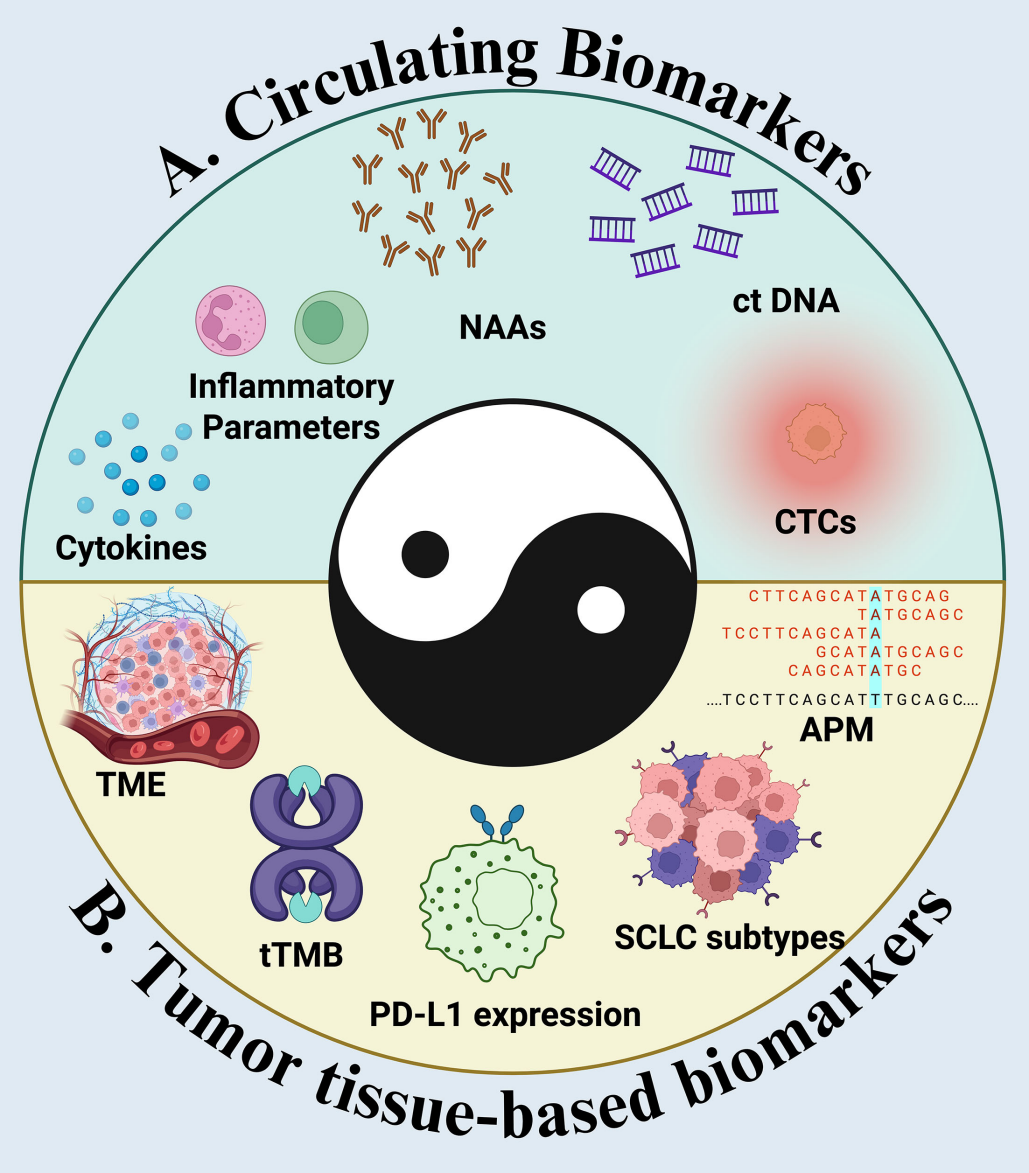
Schematic overview of complementary biomarker domains used to forecast ICI benefit in ES-SCLC. **(A)** Circulating biomarkers: dynamic ctDNA kinetics (clearance or ≥1-log decline), circulating tumor cells (CTCs) and phenotype, neoantigen-associated antibodies (NAAs), systemic inflammatory parameters (e.g., NLR, CRP), and cytokine profiles collectively index tumor burden, immune activation, and myeloid/lymphoid balance. **(B)** Tumor tissue–based biomarkers: features of the tumor microenvironment (TME) including immune exclusion or TLS signatures; tumor mutational burden (tTMB) and mutational processes; PD-L1 expression on tumor/immune cells; integrity of the antigen-processing/presentation machinery (APM) (HLA class I, B2M, TAP/TAPBP); and molecular SCLC subtypes (e.g., ASCL1-, NEUROD1-, POU2F3-, YAP1-like) that associate with distinct immune phenotypes. The central motif emphasizes that optimal prediction derives from integrating circulating and tissue signals into trajectory-based models rather than relying on any single static marker, enabling patient selection, early on-treatment adaptation, and rational trial enrichment in ES-SCLC.

Liquid biopsy approaches are transforming biomarker development. Circulating tumor DNA (ctDNA) dynamics can reflect early treatment response and predict relapse in the perioperative setting (111). Clearance of ctDNA following neoadjuvant therapy is associated with improved event-free survival, while persistent ctDNA signals minimal residual disease and high recurrence risk (112). Circulating immune cell subsets, exosomes, and cytokine profiles provide additional potential markers for predicting benefit and toxicity (**Figure 5**).

### Multi-omics and artificial intelligence

The future of biomarker development lies in integrating multi-omics data with advanced computational tools. Genomics, transcriptomics, proteomics, metabolomics, and radiomics each offer unique insights, but their combined use enables more comprehensive patient stratification. Artificial intelligence and machine learning models trained on large datasets can identify complex patterns that may be invisible to traditional analyses. While promising, these approaches must be validated in prospective trials and translated into practical clinical tools.

Single-timepoint metrics—such as PD-L1 tumor proportion score or a binary tumor mutational burden cutoff—insufficiently reflect the evolving biology of immune control in lung cancer. A trajectory-based, multimodal paradigm integrates longitudinal ctDNA kinetics, radiomic descriptors of peritumoral texture and interface entropy, peripheral immune dynamics (e.g., expansion or contraction of activated and exhausted T-cell subsets), and spatial pathology features into models that are robust in small datasets (e.g., Bayesian updating, regularized time-series deep learning) (113, 114). Rather than simply stratifying responders, the framework generates actionable probabilities—including short-term failure risk and the most likely mechanistic deficit (for example, myeloid dominance versus antigen scarcity)—to inform continuation, de-escalation, or mechanism switching. Operationalization requires standardized sampling schedules, harmonized imaging protocols, and explicit model governance to prevent “black-box” drift. Outputs should be intrinsically explainable, surfacing the signals that drive recommendations (115). Embedding this decision support within perioperative and metastatic care pathways can reduce exposure to ineffective therapy and rationalize escalation, transitioning biomarker use from static labels to real-time therapeutic navigation.

## Spatially resolved immunomics to engineer TLS-positive tumors

The prognostic and predictive value of tertiary lymphoid structures (TLSs) in lung cancer has been repeatedly observed, yet TLSs remain largely treated as passive correlates of response rather than interventional targets (107, 116). We posit a therapeutic framework in which spatially resolved immunomics—integrating multiplex histology, spatial transcriptomics, and neighborhood-level ligand–receptor inference—identifies actionable microdomains that can be rationally “converted” into TLS-positive niches (116–118). In immune-excluded NSCLC, three interdependent levers merit prospective testing: (i) terrain preparation, using vascular normalization and low-dose, hypofractionated radiation to reduce hypoxia and stromal impedance (119); (ii) localized priming, employing intratumoral oncolytic vectors, STING agonists, or dendritic-cell–targeted nanoparticles to induce B-cell/T-follicular helper clustering and to enrich antigen presentation hotspots at the invasive margin (120–122); and (iii) maintenance and maturation, coupling PD-1/PD-L1 blockade with chemokine engineering to stabilize germinal center–like architectures and sustain T-cell recruitment. In this schema, TLS maturation functions as an early pharmacodynamic endpoint, while circulating tumor DNA (ctDNA) trajectories adjudicate systemic impact (116). By turning TLSs from “signposts of immunity” into programmable bioreactors, perioperative and metastatic immunotherapy could transition from static biomarker gating to spatially guided, stage-adapted remodeling of the tumor–immune interface (123).

## Emerging roles of the airway and gut microbiome in lung cancer immunobiology

**Figure 6:**
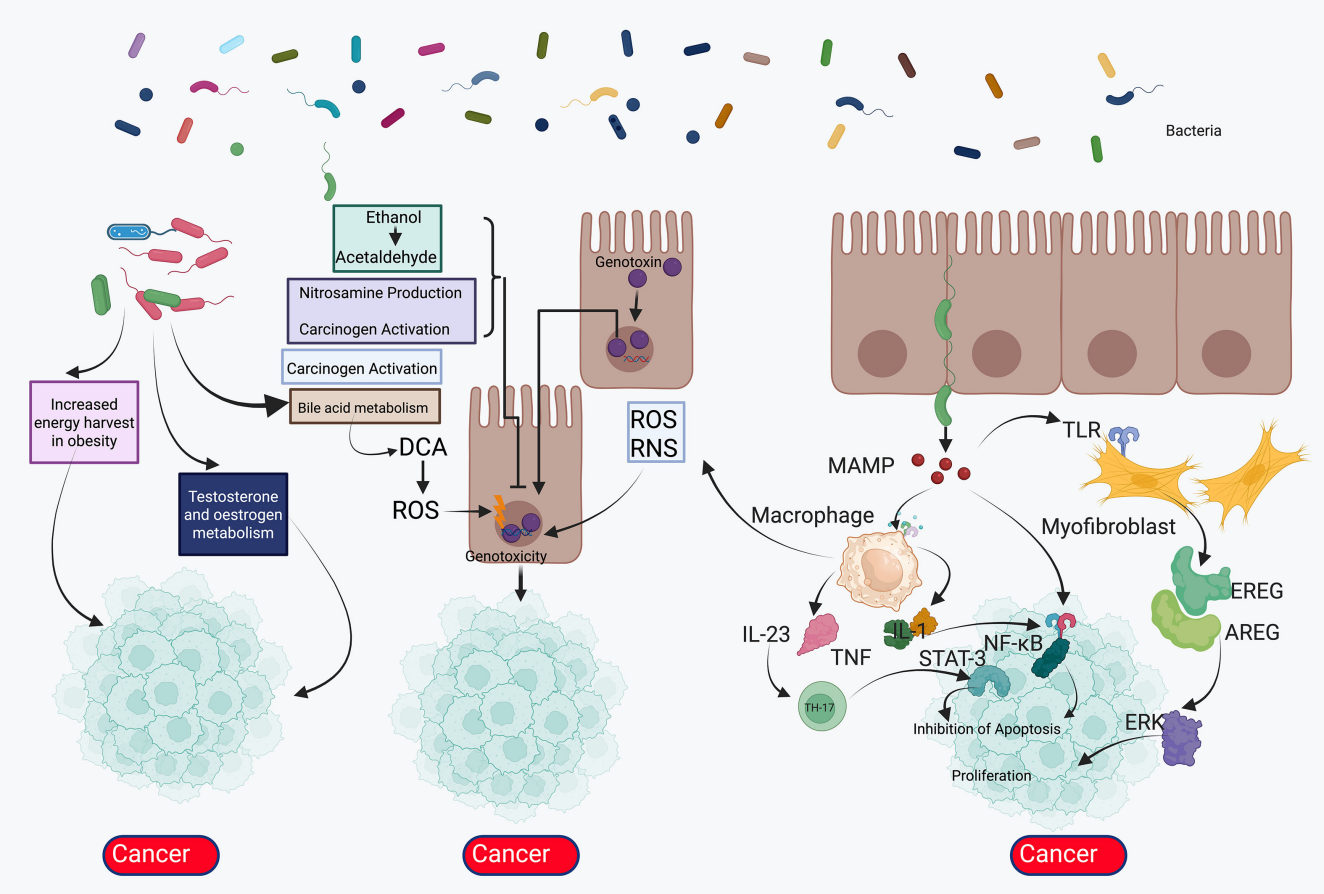
Microbiome–cancer crosstalk: metabolic, genotoxic, and inflammatory pathways driving tumor promotion. Schematic overview of microbial mechanisms that foster carcinogenesis at mucosal surfaces and within the tumor microenvironment. *Left*: Dysbiotic communities enhance caloric harvest in obesity and alter sex-hormone metabolism, indirectly supporting tumor growth. Bacterial fermentation and enzymatic activities generate ethanol/acetaldehyde, nitrosamines, and other carcinogen-activating metabolites; dysregulated bile-acid metabolism (e.g., deoxycholic acid, DCA) increases reactive oxygen species (ROS) and reactive nitrogen species (RNS), producing genotoxicity and DNA damage in epithelial cells. *Middle:* Microbe-derived genotoxins and oxidative stress propagate mutational burden and clonal selection, promoting malignant transformation. *Right:* Microbe-associated molecular patterns (MAMPs) engage toll-like receptors (TLRs) on epithelial, stromal, and immune cells, activating NF-κB and STAT3 signaling and inducing TNF and IL-23. The resulting Th17 skewing, macrophage activation, and myofibroblast remodeling (via AREG/EREG and downstream ERK) drive epithelial proliferation, survival (inhibition of apoptosis), and extracellular-matrix remodeling, thereby establishing a pro-tumor inflammatory niche. Collectively, metabolic reprogramming, DNA damage, and innate-immune activation converge to sustain tumor initiation and promotion. AREG, amphiregulin; DCA, deoxycholic acid; ERK, extracellular signal-regulated kinase; IL-23, interleukin-23; MAMP, microbe-associated molecular pattern; NF-κB, nuclear factor κB; RNS, reactive nitrogen species; ROS, reactive oxygen species; STAT3, signal transducer and activator of transcription 3; TLR, toll-like receptor; TNF, tumor necrosis factor; Th17, T helper 17.

**Figure 7:**
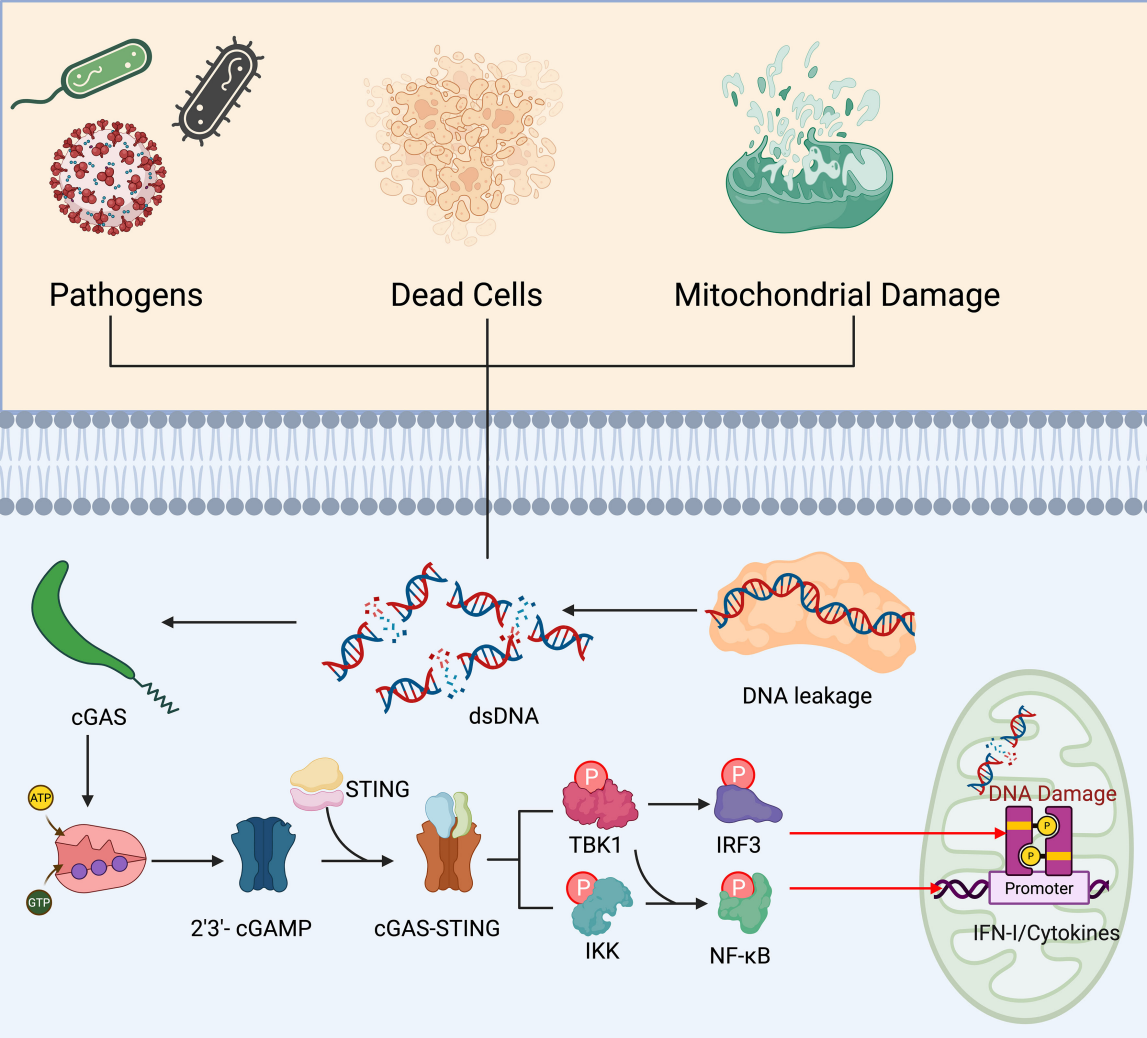
cGAS–STING–mediated tumor suppression: cytosolic DNA sensing and type I interferon induction. dsDNA arising from pathogens, dying cells, or mitochondrial damage/DNA leakage is detected by cyclic GMP–AMP synthase (cGAS), which catalyzes the formation of 2′3′-cGAMP from ATP and GTP. cGAMP engages STING on the endoplasmic reticulum and triggers STING trafficking and recruitment of TBK1 and IKK, culminating in phosphorylation and activation of IRF3 and NF-κB. The ensuing transcriptional program induces IFN-I and pro-inflammatory cytokines, enhances antigen presentation, and promotes dendritic-cell activation, cross-priming of CD8^+^ T cells, and NK-cell recruitment—collectively facilitating immune recognition and elimination of tumor cells. DNA damage within malignant cells can amplify this axis, providing a mechanistic rationale for combining cGAS–STING activation with radiotherapy, DNA-damage response inhibitors, or immune checkpoint blockade to potentiate antitumor immunity. dsDNA, Cytosolic double-stranded DNA; cGAMP, cyclic GMP–AMP; IFN-I, type I interferon; IKK, IκB kinase; IRF3, interferon regulatory factor 3; NF-κB, nuclear factor κB; STING, stimulator of interferon genes; TBK1, TANK-binding kinase 1.

The relationship between microorganisms and lung cancer is increasingly recognized as bidirectional and clinically relevant, spanning the airways, tumor bed, and the gut–lung axis (26, 124, 125). Dysbiosis of the lower airway microbiome—often characterized by enrichment of oral commensals and pathobionts with depletion of barrier-supporting taxa—can promote chronic mucosal inflammation, epithelial remodeling, and pro-carcinogenic signaling through toll-like receptors and inflammasome activation (126). Intratumoral bacteria and fungi have been detected within lung neoplasms and adjacent stroma, where they may shape the immune phenotype by skewing myeloid differentiation toward immunosuppressive macrophages and myeloid-derived suppressor cells, inducing regulatory T-cell programs, and dampening cytotoxic T-cell priming. Microbial metabolites further modulate this ecosystem: short-chain fatty acids (e.g., butyrate, propionate) can reinforce epithelial integrity and dendritic-cell tolerogenicity; indole derivatives of tryptophan signal via the aryl hydrocarbon receptor to influence Th17/Treg balance; and secondary bile acids and polyamines may foster DNA damage and tumor-promoting inflammation (127). Translocated microbial products and immune education within the intestinal mucosa condition systemic antitumor immunity. Specific commensal configurations, such as the enrichment of *Akkermansia muciniphila, Bifidobacterium* species, and *Ruminococcaceae*, have been positively associated with improved responses to immune checkpoint inhibitors. Conversely, an abundance of *Gammaproteobacteria* in the lung has been linked to poorer outcomes and resistance. In contrast, broad-spectrum antibiotics, proton-pump inhibitors, and repeated corticosteroid exposure correlate with attenuated benefit—likely by eroding microbial diversity and effector-T-cell competence (**Figure 6**). Infections and frequent antibiotic courses in patients with chronic obstructive pulmonary disease may therefore indirectly reduce immunotherapy efficacy, while radiotherapy and cytotoxic chemotherapy can remodel both airway and intestinal communities, with uncertain implications for subsequent immune responsiveness (128). The mycobiome and virome also warrant attention: fungal colonization may aggravate Th2-skewed inflammation and tissue remodeling, whereas latent or lytic viral activity can trigger cGAS–STING signaling and alter interferon tone (129) (**Figure 7**). Methodologically, the field must address low-biomass contamination, batch effects, and body-site sampling heterogeneity; integrated analyses that combine spatial microbiology, metatranscriptomics, and host single-cell profiling are essential to disentangle cause from consequence. Clinically, microbiome-informed strategies could enable risk stratification, pharmacodynamic monitoring during immunotherapy, and intervention trials testing rationally selected probiotics, postbiotics, diet and fiber modulation, targeted narrow-spectrum antibiotics, or fecal microbiota transfer—balanced against infection risk in immunosuppressed hosts (130). Collectively, these insights position the microbiome as both a modifier of lung carcinogenesis and a tunable determinant of response or resistance across the lung cancer treatment continuum.

## Combination strategies

The limited efficacy of monotherapy ICIs in many patients has driven intense exploration of combination approaches. The rationale is to overcome resistance by targeting multiple pathways simultaneously.

### Chemo-immunotherapy

Chemotherapy induces immunogenic cell death, enhances antigen release, and promotes dendritic cell activation. When combined with ICIs, these effects synergize to improve outcomes. Clinical trials have firmly established chemo-immunotherapy as the standard of care for most patients with metastatic NSCLC, regardless of PD-L1 expression (131). The durability of benefit, however, varies, and biomarkers that can guide patient selection for combination versus monotherapy remain a critical need.

### Radiotherapy plus immunotherapy

Radiotherapy is a powerful immunomodulator, which can increase antigen presentation, enhance T-cell infiltration, and induce abscopal effects. The PACIFIC trial validated sequential chemoradiotherapy followed by ICI, but there is growing interest in concurrent strategies (54, 132). Early studies suggest feasibility, though safety concerns, particularly pneumonitis, must be addressed. Optimal radiation dose and fractionation to maximize immunogenic synergy remain areas of active research.

Conventional radio-immunotherapy studies have focused largely on timing and fractionation, with limited incorporation of explicit immune objectives into dose planning (133). In immune-excluded lung tumors, a precision dose-painting approach can direct focal hypofractionated boosts to spatially defined cold niches—such as cancer-associated fibroblast–rich rims and hypoxic cores—to disrupt stromal barriers, while maintaining moderate doses across immune-hot corridors to preserve lymphocyte viability (27). Immune-informed planning should integrate radiomic signatures with spatial transcriptomic maps to delineate exclusion zones and antigen-presentation hotspots, thereby shifting from purely geometric coverage to bio-topographic remodeling. Prospective workflows ought to include near real-time biomarkers—such as post-radiation rises in circulating tumor DNA and transient expansions of activated circulating T cells—as early readouts of systemic immune activation, enabling rational escalation or de-escalation of PD-(L)1 maintenance therapy (134). Operationally, a staged strategy is appropriate, beginning with anti-angiogenic priming to normalize vasculature, followed by hypofractionated, niche-focused radiotherapy, and then PD-1 maintenance with vigilant monitoring for pneumonitis. Aligning dose distribution with immune functional goals can recast radiotherapy from a purely local cytotoxic modality into a spatially precise immune catalyst.

### Anti-angiogenic therapy plus immunotherapy

VEGF not only promotes angiogenesis but also exerts immunosuppressive effects by impairing dendritic cell maturation and recruiting Tregs and MDSCs. Anti-angiogenic agents normalize the vasculature, alleviate hypoxia, and enhance immune infiltration. The combination of anti-VEGF therapy with ICIs has demonstrated improved outcomes in NSCLC, particularly in biomarker-defined subgroups (135). Future trials are refining which patient populations derive the most benefit (136).

### Targeted therapy plus immunotherapy

The integration of targeted therapy and immunotherapy remains challenging. In EGFR- or ALK-driven NSCLC, ICIs show limited efficacy and higher rates of toxicity, particularly pneumonitis, when combined with tyrosine kinase inhibitors. In contrast, KRAS G12C inhibitors show potential synergy with ICIs, although hepatotoxicity has been a concern (101, 137, 138). Strategies such as sequencing, intermittent dosing, and rational combination with immunomodulators may help unlock the potential of targeted-ICI combinations.

### Novel agents plus immunotherapy

Novel combinations aim to overcome metabolic and immunosuppressive barriers. Adenosine pathway inhibitors, CSF1R antagonists, and STING agonists are under investigation, with the goal of reprogramming the TME (139–142). While PD-1/PD-L1 inhibition remains the cornerstone of therapy, the upregulation of alternative inhibitory receptors—co-expressed on exhausted T cells—constitutes a primary driver of adaptive resistance. To deepen clinical responses, the field is pivoting toward targeting non-redundant immune checkpoints that govern distinct phases of the immunity cycle. Foremost among these is CTLA-4, which, unlike PD-1’s primary function in the peripheral effector phase, regulates the initial priming of T cells within lymph nodes; dual blockade approaches (e.g., nivolumab plus ipilimumab) leverage this by lowering the activation threshold and depleting regulatory T cells, offering a mechanistic complement to PD-1 blockade albeit with increased toxicity (143). In the tumor microenvironment, TIGIT has emerged as a critical target due to its competition with the costimulatory receptor CD226 (DNAM-1) for PVR (CD155) binding, effectively “locking” T cells in an inhibited state (144). Although recent Phase III data (SKYSCRAPER-01) suggest TIGIT blockade may not be a universal solution, it remains a potent tool for restoring the CD226 axis in biomarker-selected populations (145). Furthermore, receptors marking specific exhaustion states offer additional avenues: LAG-3 binds to MHC Class II and FGL1 to brake proliferation in early exhaustion, a mechanism validated in melanoma and now explored in lung cancer, while TIM-3 interacts with Galectin-9 on terminally exhausted cells to induce apoptosis (146). Collectively, these emerging checkpoints represent distinct modules of immune regulation—restoring priming (CTLA-4), unlocking costimulation (TIGIT), or reversing deep exhaustion (LAG-3/TIM-3)—allowing for rational, multi-target strategies tailored to the specific exhaustion signature of a patient’s tumor. These strategies reflect a broader shift toward targeting non-T-cell components of the immune response, acknowledging that the immune ecosystem is multifaceted and requires coordinated modulation (3).

## Emerging immunotherapeutic modalities

### Adoptive cell therapy 2.0: TILs and genome-edited T cells

Unlike CAR-T cells which typically target a single surface antigen, TILs offer the distinct advantage of polyclonal recognition, targeting a diverse array of tumor-specific neoantigens. This is particularly relevant in NSCLC, which is characterized by a high tumor mutational burden and substantial antigenic heterogeneity. Following the regulatory approval of lifileucel in melanoma, the application of TILs has rapidly expanded to thoracic oncology.

The pivotal IOV-LUN-202 trial (NCT04614103) is currently evaluating LN-145 (autologous TILs) in patients with advanced NSCLC who have progressed on checkpoint inhibitors and chemotherapy (147). Early data suggests that TILs can induce durable responses even in PD-1 refractory settings, likely by leveraging T-cell clones that recognize cryptic or private neoantigens ignored by previous therapies. However, challenges remain regarding the complex manufacturing process and the need for lymphodepletion, which limits accessibility for frail patients. Future iterations utilizing “genetically enhanced” TILs—engineered to secrete IL-2 or resist TGF-β suppression—are under investigation to improve persistence and reduce systemic toxicity.

### CRISPR-Cas9 and genome editing strategies

The efficacy of adoptive T-cell therapy is often curtailed by T-cell exhaustion and the immunosuppressive TME. CRISPR-Cas9 gene editing technology provides a powerful toolkit to overcome these barriers by precisely deleting inhibitory checkpoints or reinforcing effector functions (148).

A landmark proof-of-concept study demonstrated the feasibility of multiplex CRISPR-Cas9 editing in patients with advanced cancer. In this approach, T cells were engineered to express a TCR targeting the cancer-testis antigen NY-ESO-1, while simultaneously disrupting the PDCD1 (encoding PD-1) and TRAC (T-cell receptor α constant) genes (149, 150) The deletion of endogenous TCR prevents mispairing and autoimmunity, while the knockout of PD-1 shields the engineered cells from checkpoint inhibition. These “insulation” strategies have shown potential to extend T-cell persistence and maintain cytotoxicity within the hostile tumor bed. Moving forward, next-generation editing, including base editing and prime editing, promises to enable even more complex reprogramming—such as “armoring” T cells against metabolic stress or converting inhibitory signals (e.g., FAS or TIGIT) into activating ones—marking the dawn of synthetic immunity in lung cancer (151, 152).

### Bispecific T-cell engagers

Bispecific antibodies that simultaneously bind tumor antigens and CD3 on T cells represent a new frontier. In SCLC, DLL3-targeted bispecifics have demonstrated clinical activity and recently gained regulatory approval for relapsed disease (80). These agents overcome the need for pre-existing T-cell infiltration by directly redirecting T-cell cytotoxicity toward tumor cells. In NSCLC, bispecifics targeting EGFR and MET (e.g., amivantamab) are already approved in molecularly defined subsets, highlighting the versatility of this platform (153, 154).

### CAR-T and TCR-T cell therapies

CAR-T cells have achieved remarkable success in hematologic malignancies but face significant barriers in solid tumors (155–157). Antigen heterogeneity, poor trafficking, and hostile TMEs limit efficacy. Early-phase trials in NSCLC targeting MUC1, mesothelin, and EGFR variants show feasibility but modest activity (158–160). Strategies to enhance persistence and function, such as armored CARs and logic-gated CARs, are being explored (161, 162). TCR-engineered T cells, which recognize intracellular antigens presented by HLA molecules, provide another avenue (163). Targeting shared neoantigens such as KRAS mutations offers opportunities for broader application (**Table 3**).

### NK-cell and macrophage-based therapies

Natural killer cells offer advantages as allogeneic, off-the-shelf products. CAR-NK cells can target tumor antigens without the risk of graft-versus-host disease and may induce fewer cytokine release syndromes (164). CAR-macrophages, meanwhile, are designed to reprogram the TME by enhancing phagocytosis and antigen presentation (165). Although still early in development, these platforms represent promising complements to T-cell.

### Cytokine-based therapies

Cytokines such as IL-2 and IL-15 are potent immune activators, but native forms are limited by toxicity and expansion of Tregs (24, 166). Next-generation cytokine agonists are engineered to selectively stimulate cytotoxic lymphocytes while minimizing side effects (167, 168). Early trials in solid tumors, including lung cancer, show encouraging immune activation, particularly when combined with ICIs. These agents could play an important role in broadening the reach of immunotherapy.

### Oncolytic viruses and cancer vaccines

Oncolytic viruses selectively infect and lyse tumor cells, releasing antigens and inducing immunogenic cell death (169–172). They can also be engineered to express cytokines or checkpoint inhibitors. Therapeutic vaccines targeting tumor-associated antigens or personalized neoantigens are another strategy to enhance immune recognition (173). While results have been modest in unselected lung cancer populations, combining vaccines or oncolytic viruses with ICIs holds promise. Advances in mRNA vaccine technology have accelerated this field, with ongoing trials evaluating lung cancer–specific applications (174–176).

## Preclinical models and translational tools

Robust preclinical models are essential for translating immunotherapy discoveries into clinical practice. Genetically engineered mouse models (GEMMs) of lung cancer, such as those driven by Kras and p53 alterations, provide platforms for studying immune interactions in an immune-competent setting (177, 178). Syngeneic models allow evaluation of immunotherapy combinations, although they may not fully recapitulate human tumor heterogeneity.

Patient-derived xenografts (PDXs) and organoids provide valuable insights but lack a complete immune system, limiting their utility for immunotherapy research (179). Humanized mouse models, which incorporate human immune cells, address this limitation but remain costly and technically challenging (177, 180). Advances in spatial transcriptomics, multiplex immunohistochemistry, and single-cell sequencing have provided unprecedented resolution of the TME, enabling detailed mapping of immune ecosystems. These tools are critical for identifying novel targets, validating biomarkers, and guiding rational trial design.

## Toxicity and management

Immunotherapy is associated with unique toxicities that differ from chemotherapy and targeted therapy (181). Immune-related adverse events arise from immune activation against normal tissues and can affect any organ system (182). In lung cancer, pneumonitis is particularly significant, given the background of thoracic disease and prior radiation exposure (183). Early symptoms such as cough and dyspnea require prompt recognition, as severe cases can be life-threatening. Management typically involves corticosteroids and treatment interruption, with multidisciplinary input from oncology, pulmonology, and radiology.

Other irAEs include dermatitis, colitis, hepatitis, and endocrinopathies (184, 185). For bispecific antibodies and CAR-based therapies, cytokine release syndrome and neurotoxicity are major concerns, necessitating specialized monitoring and supportive care protocols (186, 187). Proactive toxicity management is essential to maximize the benefit of immunotherapy without compromising patient safety.

As immunotherapy moves into earlier stages of disease and combination regimens, toxicity profiles become more complex. Developing predictive biomarkers for toxicity and standardizing management protocols will be critical to safely expanding the reach of immunotherapy.

## Equity, access, and regulatory considerations

The promise of lung cancer immunotherapy is tempered by inequities in access (188). Even within high-income nations, disparities exist along racial, socioeconomic, and geographic lines. Globally, the divide is even more pronounced. Recent analyses of the clinical trial landscape reveal that over 60% of immunotherapy trials are concentrated in North America and Western Europe, with East Asia (particularly China) rapidly emerging as a third dominant hub. In stark contrast, low- and middle-income countries LMICs—which bear a rising proportion of the global lung cancer burden—remain on the periphery of innovation. For instance, data presented at major oncology forums indicate that less than 3% of global immunotherapy trials include sites in Sub-Saharan Africa or lower-middle-income Southeast Asian nations. This geographic skewing creates a ‘data desert,’ leaving uncertainty about the efficacy and toxicity of these agents in diverse genetic backgrounds. The barriers preventing equitable access are multifaceted, ranging from the lack of high-fidelity biomarker testing infrastructure (e.g., NGS, PD-L1 IHC) to the prohibitive costs of cold-chain logistics and the drugs themselves.

Regulatory agencies face the challenge of balancing rapid approval of promising agents with the need for confirmatory evidence. Accelerated approvals based on surrogate endpoints have expedited access but require rigorous follow-up. Ethical considerations include the inclusion of diverse populations in clinical trials, affordability of novel therapies, and transparency in regulatory decisions (189).

Addressing these challenges will require global collaboration, health policy innovation, and advocacy to ensure that the benefits of immunotherapy are realized broadly, not just in privileged populations.

## Future directions and perspectives

The future of lung cancer immunotherapy is poised to be defined by precision, integration, and accessibility. First, personalization will deepen, with ctDNA minimal residual disease monitoring and multi-omic biomarkers guiding tailored perioperative therapy. Second, rational combination strategies that target metabolic, stromal, and myeloid compartments will seek to convert immune-excluded tumors into responsive ones. Third, emerging modalities such as bispecifics, CAR-based therapies, and vaccines will expand the therapeutic arsenal beyond PD-1/PD-L1 blockade. Fourth, preclinical and translational tools will continue to refine understanding of tumor–immune interactions, enabling more efficient trial design and biomarker validation. Fifth, managing toxicity will remain critical as regimens become more intensive and complex. Finally, global equity and access must remain a priority, ensuring that immunotherapy is not limited to select populations. Collectively, these directions signal an era in which lung cancer immunotherapy evolves from broad checkpoint inhibition to integrated, biomarker-informed, and stage-specific strategies capable of delivering durable remissions and cures.

## Conclusions

Lung cancer immunotherapy has undergone a remarkable transformation, moving from experimental salvage therapy to a central component of care across all stages of disease. Yet challenges remain: resistance limits efficacy, toxicity complicates management, and inequities in access persist. The field is advancing toward solutions through the development of multi-layered biomarkers, rational combinations, and novel therapeutic modalities. By integrating these approaches with robust translational research and global health strategies, the next decade of lung cancer immunotherapy holds the potential to substantially reduce the burden of this devastating disease.
